# Nutritional Approaches for the Management of Metabolic Acidosis in Chronic Kidney Disease

**DOI:** 10.3390/nu13082534

**Published:** 2021-07-24

**Authors:** Annalisa Noce, Giulia Marrone, Georgia Wilson Jones, Manuela Di Lauro, Anna Pietroboni Zaitseva, Linda Ramadori, Roberto Celotto, Anna Paola Mitterhofer, Nicola Di Daniele

**Affiliations:** 1 UOC of Internal Medicine-Center of Hypertension and Nephrology Unit, Department of Systems Medicine, University of Rome Tor Vergata, 00133 Rome, Italy; dilauromanuela@gmail.com (M.D.L.); annapietroboni@icloud.com (A.P.Z.); linda.ramadori@gmail.com (L.R.); annapaola.mitter@uniroma2.it (A.P.M.); didaniele@med.uniroma2.it (N.D.D.); 2 Center of Research of Immunopathology and Rare Diseases—Nephrology and Dialysis Coordinating Center of Piemonte and Aosta Valley Network for Rare Diseases, S. Giovanni Bosco Hospital, Department of Clinical and Biological Sciences, University of Turin, 10154 Turin, Italy; georgia.wilson.jones@gmail.com; 3 School of Specialization in Geriatrics, University of Rome Tor Vergata, Via Montpellier 1, 00133 Rome, Italy; 4 Department of Cardiovascular Disease, Tor Vergata University of Rome, 00133 Rome, Italy; celott.roberto@yahoo.it

**Keywords:** chronic kidney disease, metabolic acidosis, vegan diet, low-protein diet, nutritional therapy, PRAL

## Abstract

Metabolic acidosis is a severe complication of chronic kidney disease (CKD) which is associated with nefarious impairments such as bone demineralization, muscle wasting, and hormonal alterations, for example, insulin resistance. Whilst it is possible to control this condition with alkali treatment, consisting in the oral administration of sodium citrate or sodium bicarbonate, this type of intervention is not free from side effects. On the contrary, opting for the implementation of a targeted dietetic-nutritional treatment for the control of CKD metabolic acidosis also comes with a range of additional benefits such as lipid profile control, increased vitamins, and antioxidants intake. In our review, we evaluated the main dietary-nutritional regimens useful to counteract metabolic acidosis, such as the Mediterranean diet, the alkaline diet, the low-protein diet, and the vegan low-protein diet, analyzing the potentialities and limits of every dietary-nutritional treatment. Literature data suggest that the Mediterranean and alkaline diets represent a valid nutritional approach in the prevention and correction of metabolic acidosis in CKD early stages, while the low-protein diet and the vegan low-protein diet are more effective in CKD advanced stages. In conclusion, we propose that tailored nutritional approaches should represent a valid therapeutic alternative to counteract metabolic acidosis.

## 1. Introduction

The German philosopher Ludwig Feuerbach put forward the concept of “you are what you eat” in 1863 [1]. These words hold true now more than ever. Currently, researchers and physicians are delving deep into the exploration of the beneficial potentials of dietary intervention in the treatment of chronic non-communicable diseases (NCDs). Amongst the latter, we find chronic kidney disease (CKD), a condition characterized by persistent loss of kidney function, urinary abnormalities and renal parenchyma alterations. Clinically, it can be defined as a glomerular filtration rate (GFR) <60 mL/min/1.73 m^2^ and/or albuminuria ≥30 mg/g of creatinine, present for more than 3 months [2]. If left unchecked, CKD can evolve into end-stage renal disease (ESRD), or the need for renal replacement therapy (RRT) [3]. Additionally, CKD leads to the development of a plethora of cardiovascular, gastro-enteric, nervous, metabolic, and hormonal alterations. More common complications include hyperkalaemia, metabolic acidosis, calcium-phosphorus metabolism impairment, water and sodium metabolism alterations, changes in the composition of the gut microbiota, oxidative stress, hyperhomocysteinemia, chronic low-grade inflammation, and normocytic normochromic anaemia [2,4,5,6,7,8,9,10,11]. CKD imposes a major burden on global health, not only because it is a direct cause of morbidity and mortality, but also as a key risk factor for cardiovascular disease (CVD). In 2017, it was estimated that 700 million people were suffering from CKD, which resulted in 1.2 million deaths worldwide [12]. Current management of CKD includes strategies aimed at early detection and prevention, possible treatment of the underlying cause, and preservation of residual renal function via blood pressure control through the inhibition of the renin–angiotensin-aldosterone system (RAAS) [13]. Eventual cardiovascular comorbidities should be treated by keeping the lipid profile in check with diet and lipid-lowering drugs, decreasing blood pressure values, regulating calcium-phosphorus metabolism impairments, counteracting metabolic acidosis, and reducing albuminuria [2]. According to the 2020 Kidney Disease Outcome Quality Initiative (K/DOQI), besides the pharmacological therapy, the nutritional treatment plays a pivotal role in the management of CKD [14,15].

Given the epidemiological importance of CKD and its morbidity and mortality, it is of utmost relevance to research novel approaches to prevent it and control its progression. The present review sets out to elucidate the ways in which this can be done by using different nutritional-dietary regimens potentially useful for the metabolic acidosis management in CKD patients [14,16,17].

## 2. Metabolic Acidosis

Metabolic acidosis is a condition in which the organism’s acid–base balance is jeopardized, characterized by the presence of hydrogen ions excess resulting in the acidification of the blood’s pH [18]. Clinically, metabolic acidosis can be defined in the first instance by a serum bicarbonate concentration <22 mmol/L and, in the second instance, by a decrease in arterial partial pressure of carbon dioxide (PaCO_2_) [18]. The kidneys play a major role in the regulation and maintenance of the acid–base homeostasis, preventing metabolic acidosis by regenerating bicarbonate ions and eliminating the excess of hydrogen ions [19]. In fact, physiologically, the kidney contributes to the maintenance of acid–base balance in three ways: (i) neutralization of acids; (ii) excretion of acids; and (iii) excretion of anions [20]. With the CKD progression, the kidney’s ability to neutralize and eliminate acids is substantially reduced [21].

**Figure 1:**
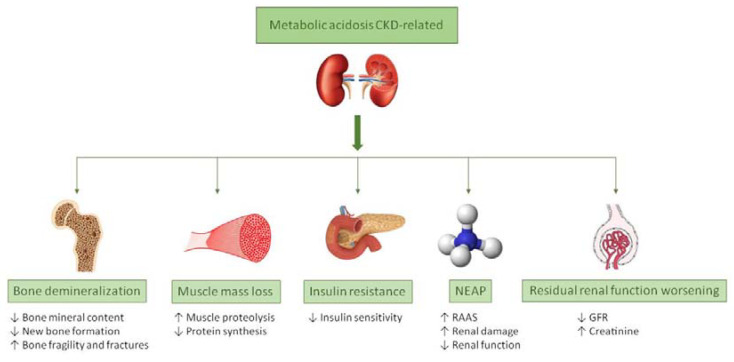
Main consequences of metabolic acidosis in CKD. Abbreviation: CKD, Chronic kidney disease; GFR, glomerular filtration rate; NEAP, net endogenous acid production; RAAS, renin–angiotensin-aldosterone system.

Therefore, a chronic reduction in GFR, above all when it is lower than 30 mL/min/1.73 m^2^, is an important risk factor for the development of metabolic acidosis. Given the association between metabolic acidosis and pathologic processes such as bone demineralization, muscle proteolysis, insulin resistance, poor ability to counteract net endogenous acid production (NEAP) (Figure 1), faster CKD progression, and increased mortality risk, it is of utmost importance to find effective ways of controlling this condition [22]. In fact, counteracting metabolic acidosis helps to preserve muscle mass and to improve bone metabolism [23,24,25,26]. An adjuvant treatment to pharmacological therapy in metabolic acidosis management is nutritional therapy. The consumption of different dietary components affects the body’s acid–base balance. For instance, meat, fish, cheese, and other foods rich in animal-derived protein favor the generation of hydrogen ions. On the contrary, fruit and vegetables favor the production of bases, counteracting hydrogen ion excess, thus having an alkalizing potential. More specifically, acid releasing dietary components are compounds containing phosphorus and proteins (i.e., sulfur amino acids, such as cysteine, methionine and taurine, and cationic amino acids such as lysine and arginine), whereas, alkalinizing ones are potassium, magnesium, and calcium [27].

The net dietary acid load is dictated by the balance between acid-forming and base-forming precursors present in the diet. Generally, diets with a high intake of animal-origin foods induce high levels of NEAP, compared to plant-based diets which result in low NEAP [28]. The typical Western diet is characterized by a high acid load with positive NEAP. This diet, which is common to Westernized countries, is made up by a high intake of saturated fats and simple sugars coupled with a low intake of fiber [29]; it is also strongly correlated with the occurrence of obesity and other metabolic diseases. The pathophysiologic mechanisms which underlie this correlation are linked to an increased production of reactive oxygen species (ROS) that causes oxidative stress, insulin resistance, arterial hypertension and chronic low-grade inflammation [30,31,32,33,34].

An interesting concept put forward by Cordain et al. compares the dietary regimens of ancestral humans (plant-based, with high levels of fiber and potassium, and low levels of refined carbohydrates and sodium) to the modern Western diet. The authors discuss the idea of an “evolutionary collision” between the ancient human genome encoding core metabolic machinery and the nutritional qualities of the contemporary energy-dense and nutrient-poor diet. They postulate that, on an evolutionary scale, this change has occurred too rapidly, resulting in a mismatch which may underlie many of the NCDs typical of the Western civilization [35].

The potential renal acid load (PRAL) can be defined as the capacity of acid or base production of any food [27], which can be calculated from the dietary intake. If the PRAL value is negative, the food has base-forming potential, whereas if it is positive, the food has acid-forming potential. For example, fruit and fruit juices have a PRAL of −3.1 (mEq/100 g) compared to meat and processed meats which have a PRAL of +9.5 (mEq/100 g) [36].

### 2.1. Metabolic Acidosis and Bone Demineralization

Metabolic acidosis determines important repercussions on the bone. In addition to secondary hyperparathyroidism and to the accumulation of uremic toxins, metabolic acidosis is an important risk factor for the onset of bone metabolism alterations in CKD patients [37]. In the presence of metabolic acidosis, one of the first lines of defense designed to block changes in blood pH is represented by the mobilization of sodium, potassium, and carbonate together with calcium, at the level of the bone tissue. This leads to a reduction in bone mineral content which causes an increase in bone fragility and an enhanced risk of fractures [38]. Furthermore, metabolic acidosis inhibits the formation of new bone tissue. As shown in vitro, metabolic acidosis appears to inhibit the activity of osteoblasts and increase the activity of osteoclasts [39]. On the contrary, an increase in the concentration of serum bicarbonate associates with a reduction in osseous calcium efflux and an increase in osteoblastic activity [40]. Metabolic acidosis would also seem to inhibit osteoblast differentiation [41].

Furthermore, metabolic acidosis appears to modulate gene expression at the level of bone cells, specifically by inducing a reduction in the expression of the genes responsible for the synthesis of type I collagen fibrils in osteoblasts [42]. Finally, metabolic acidosis induces an increase in prostaglandin production, which are powerful regulators of bone metabolism. In particular, it has been shown that an increase in prostaglandin concentration, in particular prostaglandin E2, leads to an enhancement in the outflow of calcium from the bones [43].

After 10 weeks of induction of metabolic acidosis by administration of NH_4_Cl, Gasser et al., observed a reduction in total volumetric bone mineral density and in trabecular bone volume in animal models. These results confirmed that metabolic acidosis can inhibit bone formation whilst stimulating bone resorption [44].

### 2.2. Metabolic Acidosis and Muscle Mass Wasting

CKD patients are characterized by skeletal muscle tissue loss related to the protein-energy wasting (PEW) syndrome, to chronic low-grade inflammation, and to the onset of metabolic acidosis which stimulates an increase in muscle proteolysis. All these factors contribute to accelerate the muscle mass loss leading to uremic sarcopenia [25].

Several studies suggest that metabolic acidosis is able to stimulate the proteolysis of skeletal muscle mass. Specifically, Mitch et al. highlighted how metabolic acidosis can inhibit the ATP-ubiquitin-proteasome system increasing the expression of the genes that code for ubiquitin and for other proteolytic subunits of the proteasome [45,46]. In animal models, acidosis appears to be associated with a 2.5-to-4-fold increase in ubiquitin mRNA in muscle which appears to normalize 24 h after acidosis correction, confirming the role of the ubiquitin-proteasome pathway in inducing proteolysis [45]. Furthermore, metabolic acidosis is able to inhibit, even if only minimally, muscular protein synthesis [47]. Another possible indirect mechanism by which metabolic acidosis stimulates the loss of muscle mass is through the induction of insulin resistance. In fact, metabolic acidosis is often associated with defects in the insulin signaling which can lead to the loss of muscle mass [48,49].

These findings have also been confirmed in clinical studies. In a recent study conducted on 188 CKD patients with metabolic acidosis (bicarbonate values < 22 mmol/L), Dubey et al. demonstrated that the treatment of metabolic acidosis by oral sodium bicarbonate supplementation increased mid-arm muscle circumference and lean mass, as well as ameliorating the kidney function. These results suggest that the resolution of metabolic acidosis improves muscle mass growth and increases protein reserves in CKD patients [24].

### 2.3. Metabolic Acidosis and Insulin Resistance

The establishment of metabolic acidosis leads to important hormonal alterations, including impairment in parathyroid hormone balance, insulin-like growth factor 1 (IGF-1) signaling, insulin secretion, thyroid hormone, and glucocorticoid hormone levels [50]. Specifically, metabolic acidosis has been associated with alterations of glucose homeostasis and with the reduction in insulin sensitivity [51]. Several clinical studies have highlighted important correlations between insulin resistance and metabolic acidosis such as lower bicarbonate levels [52] and urine pH [53]. Animal studies have shown that metabolic acidosis seems to inhibit the uptake and oxidation of glucose inducing an impaired insulin sensitivity [54]. Oral sodium bicarbonate supplementation in CKD patients with metabolic acidosis appeared to increase insulin sensitivity [55]. In fact, in a recent study conducted on CKD patients with metabolic acidosis, an improvement in the homeostatic model assessment (HOMA) index was observed after oral sodium bicarbonate assumption up to restore physiological pH values [56].

### 2.4. Metabolic Acidosis and NEAP

Metabolic acidosis involves high levels of NEAP, which can have important clinical relapses, especially in the kidney [57]. In particular, metabolic acidosis and a NEAP increase can lead to an increase in RAAS activity [58]. This increase is associated with renal damage and an acceleration in renal functional decline [59,60]. The resolution of metabolic acidosis can reverse this process by reducing aldosterone and angiotensin II production [61].The latter is an important pro-inflammatory and pro-fibrotic agent that contributes to CKD progression [60].

Furthermore, high levels of NEAP increase endothelin production [62,63] that induces vasoconstriction, inflammation, and fibrosis. The suppression of endothelin is associated with the reduction in blood pressure and proteinuria [64].

In addition to the faster progression of kidney damage and to the greater loss of function, high NEAP levels are associated with an increased risk of developing CKD, as demonstrated in healthy subjects with high NEAP levels [65].

### 2.5. Metabolic Acidosis- Induced Kidney Injury

The onset of metabolic acidosis in CKD induces a series of compensation mechanisms aimed at increasing acid excretion, leading to the worsening of residual kidney function [66]. Metabolic acidosis in CKD stimulates intrarenal production of hormones such as angiotensin II, aldosterone, and endothelin-1, which increase renal acid excretion [63,67]. These hormones enhance the transport of hydrogen ions in the proximal tubule and the proton-translocating ATPase (H+ -ATPase), inducing a rapid decline in residual renal function. Hormones released in the kidney cause direct damage to the renal parenchyma by promoting local inflammation and the fibrotic process, which, in turn, are aggravated by the continuous stimulation of ammoniagenesis. The latter damages the kidney through complement activation. Specifically, angiotensin I is converted into angiotensin II in the distal tubules, where it promotes tubular acidification and tubular acid secretion through an enhanced expression of Na-hydrogen exchanger 3 [68]. This may trigger interstitial inflammation, fibrosis and tubular atrophy, accelerating the progression of CKD. In CKD patients, an increased intrarenal production of endothelin-1 is observed; it induces the distal tubular secretion of H+. This also seems to induce an increased tubular-interstitial damage, related to the local inflammation and fibrosis [69]. These contribute to increasing glomerular permeability and consequently the decline of residual renal function. Finally, the intrarenal stimulation of the RAAS, due to an increased acid retention, can lead to an up-regulated production of aldosterone which induces an increase in the acidification of the distal nephrons and promotes renal damage [70].

The treatment of chronic metabolic acidosis in CKD patients, both pharmacological and nutritional, would seem to attenuate the maladaptive responses of the kidneys by reducing the levels of angiotensin II, aldosterone, endothelin-1 and ammoniagenesis and whilst simultaneously slowing down CKD progression. The most effective nutritional treatments in counteracting metabolic acidosis related to CKD are described below in detail.

## 3. Dietary Approaches to Counteract Metabolic Acidosis in CKD

**Figure 2:**
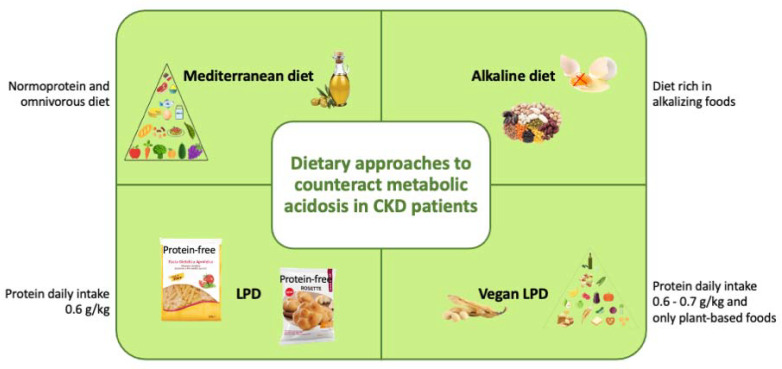
Dietary approaches to counteract metabolic acidosis in CKD patients. Abbreviations: b.w, body weigh; LPD, low-protein diet.

Modulating the type and quality of diet is pivotal for CKD management. Due to kidney damage, CKD patients exhibit impaired toxin and nitrogen waste product elimination [14]. The main dietary approaches to counteract metabolic acidosis in CKD patients are illustrated in Figure 2.

One of these is the Mediterranean diet (MD), effective in the prevention and treatment of early CKD (stages I-IIIa) [17,71,72].

Another innovative dietetic approach for CKD early stage is the alkaline diet (AD), which is constructed based on PRAL and potassium content present in different food types [73]. This dietetic treatment, rich in fruit and vegetables, can represent a valid therapeutic alternative to traditional pharmacological therapy based on bicarbonate assumption. Other positive characteristics of the AD are represented by its low cost and the variety of foods [74].

The mainstay nutritional therapy for CKD patients under conservative therapy is dietary protein restriction [75]. The low-protein diet (LPD) is characterized by a protein intake lower than 0.8 g/kg of b.w. per day with an energy intake between 25–35 kcal/kg of b.w. per day [76]. The mechanism of action of LPDs relies on the fact that a lower protein intake will result in the production of less protein-derived waste products coupled with diminished retention of fixed acids. By reducing hyperfiltration and proteinuria in the residual nephron, the progression of CKD is slowed down and the start of RRT can ideally be delayed [77]. This has been confirmed in an Italian study conducted by Bellizzi et al. which highlighted that a LPD was able to reduce the progression of CKD through the reduction in proteinuria, the improvement of blood pressure levels, and the correction of metabolic acidosis [75]. There are different types of LPDs. The vegan LPD, for instance, provides 0.6 to 0.7 g/kg of b.w. per day. Cupisti et al. defined the vegan LPD as a low-phosphorus diet based on strict combinations of cereals and legumes to provide the essential amino acids as an option for patients who refuse the use of protein-free products or when they result unavailable [14]. A vegan LPD is set up by encouraging the consumption of plant-based foods such as legumes, vegetables, cereals, and tubers. Following a vegan LPD means to implement the intake of healthy nutrients such as vitamins, fibers, natural bioactive compounds, for example, minor polar compounds (MPCs) present in extra virgin olive oil (EVOO), and antioxidants present in plants-based foods, and to simultaneously remove harmful phosphate additives from animal-derived ultra-processed foods. This reflects positively on the control of CKD related comorbidities such as arterial hypertension, hyperphosphatemia, metabolic acidosis, and dyslipidaemia [14]. Harnessing the alkalinizing potential of a plant-based diet, especially in the CKD patients, can help in the prevention of metabolic acidosis and its deleterious consequences.

### 3.1. Mediterranean Diet

The MD represents a healthy eating pattern associated with numerous beneficial effects and correlated with the reduction in the risk of developing NCDs. Numerous studies suggest that the MD can exert a positive impact on the onset and progression of NCDs [17,71,72,78,79,80,81,82,83,84,85]. The role played by the consumption of a MD in preserving the state of individual health, and in positively impacting on longevity, was definitively sanctioned in 2010 by United Nations Educational, Scientific and Cultural Organization (UNESCO), defining it “an intangible cultural heritage of humanity” [86].

The MD is characterized by the regular consumption of plant-based foods such as fruit, vegetables, legumes, cereals and nuts, and the use of EVOO. It is also known for being the main source of vegetal fats and for allowing a moderate consumption of red wine [87,88]. The consumption of EVOO and nuts makes the MD rich in monounsaturated fatty acids (MUFAs) and polyunsaturated fatty acids (PUFAs), while maintaining a low intake of saturated fatty acids (SFAs) deriving from foods such as dairy products and meat [79]. Both MUFAs and PUFAs are associated with numerous health benefits [89,90,91]. In addition, the MD is rich in foods with high content of antioxidant and anti-inflammatory compounds, capable of preventing various diseases related to oxidative stress and chronic inflammation state. Amongst these foods, EVOO (rich in polyphenols and MPCs) induces several protective effects in CKD patients, counteracting oxidative stress, inflammation, and improving purine and lipid metabolism [92,93]. Furthermore, these effects are maintained over time, as reported in our recent clinical study [94].

Several studies have been conducted on CKD patients under conservative therapy in order to evaluate the therapeutic efficacy of this diet. Our study was conducted on 50 male CKD patients (stages II-III according to K/DOQI guidelines) and 100 healthy subjects. The enrolled subjects followed an Italian MD (IMD) for 14 days and for the following 14 days an Italian Mediterranean Organic Diet (IMOD). Both dietary plans were elaborated according to Nicotera guideline [95], but in the IMD period, the enrolled subjects consumed conventional foods, while during IMOD period, they consumed organic foods. At the end of the study, CKD patients showed a reduction in homocysteine, microalbuminuria, total cholesterol, and phosphorus concentration coupled with an increase in vitamin B12 levels. Moreover, CKD patients presented a reduction in fat mass and an improvement in lean mass monitored by Dual X-ray Absorptiometry (DXA) [84]. A subsequent study by the same authors showed that IMD and IMOD represent a valid nutritional strategy alternative to LPD in the treatment of CKD in its early stages [17].

Along with CKD progression, alterations of mineral metabolism may appear, contributing to the onset of metabolic acidosis. Therefore, a diet rich in acidifying foods (high in hydrogen ions) containing phosphorus and proteins of an animal-origin, such as eggs, cheese, and meat, can alter the blood pH. A possible remedy for this could be the regular consumption of alkalizing foods, rich in potassium, calcium, and magnesium, such as fruit, vegetables, whole grains, legumes, and nuts. These foods characterize the MD [96,97] and are potentially useful in treating metabolic acidosis. However, in the CKD, more advanced stages are necessary to decrease the consumption of foods rich in potassium, as stages IV-V of CKD are characterized by low potassium excretion and hyperkalaemia [76,98]. In this regard, patients should be educated to cook vegetables by boiling them, the vegetables should be boiled twice or for a long time before consumption. Moreover, fruit intake should be limited to a maximum of 300 g per day, and low-potassium content fruit (such as apples, pears, strawberries, etc.). should be favored, whilst fruit with high-potassium content (such as bananas, peaches, etc.) should be avoided. It is also necessary to pay attention to hidden sources of potassium, such as food additives in preserved foods [99].

If the physiological pH range cannot be reached with the nutritional-dietary treatment, the assumption of sodium bicarbonate (NaHCO_3_) is recommended. The advised dose of NaHCO_3_ is 0.5 to 1.0 mEq of NaHCO_3_/kg of b.w. per day in patients with serum [HCO_3_] < 22 mmol/L [100]. A randomized clinical trial conducted on 76 hypertensive patients with CKD stage IV demonstrated that, after a one-year treatment, the dietary reduction in the acid load, related to an increased consumption of fruit and vegetables or to the oral administration of sodium bicarbonate, were both able to increase plasma bicarbonate values compared to baseline values with metabolic acidosis improvement. All enrolled patients presented a low risk of hyperkalemia, as the exclusion criterion of the study was a very low GFR. The study highlighted equivalent therapeutic efficacy between the increased intake of fruit and vegetables and an oral intake of sodium bicarbonate [101].

In summary, the MD can be an effective nutritional dietary strategy for the treatment of metabolic acidosis in the early stages of CKD. However, in the more advanced stages, it is necessary to control the potassium intake to avoid hyperkalemia. In fact, in moderate–severe CKD, the LPD or vegan LPD nutritional treatments would seem to be more effective [77].

### 3.2. Alkaline Diet

Alkali treatment consists in the administration of sodium citrate or sodium bicarbonate to patients with serum bicarbonate levels below 22 mmol/L (as recommended by K/DOQI guidelines) [100]. Such therapy is aimed to increase bicarbonate levels and to delay GFR decline. However, this type of intervention is not free from side effects. In fact, sodium citrate can increase gastric aluminum absorption and sodium bicarbonate can cause bloating and flatulence. Moreover, sodium containing compounds can enhance water retention and, therefore, contribute to arterial hypertension [74]. Having reviewed these side effects, opting for a non-sodium-based therapy and administrating a dietary plan apt to control metabolic acidosis may prove to have more beneficial effects for CKD patients. Therefore, harnessing the alkalinizing potential of a plant-based diet is a useful tool for the CKD metabolic acidosis management.

The agricultural and industrial revolutions have shaped the modern diet of Western countries. Industrialization of food has led to a decrease in potassium content, coupled with an increase in sodium and chloride content in the diet. Such a shift in dietary chemical composition has translated into a more acidic diet which may favor the induction of metabolic acidosis [102]. On average, a normal kidney can excrete 45 mEq H^+^ per day whilst a diseased kidney can only excrete about 20 mEqH^+^ per day. However, a typical Western diet produces 50 to 100 mEqH^+^ per day [73]. This mismatch between the amount of acids introduced in the organism and the ones effectively excreted causes the accumulation of acids in human organism, with consequent metabolic acidosis. Since the consumption of proteins leads to a higher production of acid and the consumption of fruit and vegetables leads to a higher production of alkali species, the AD represents a nutritional approach helpful to counteract the metabolic acidosis [103].

The AD is characterized by a high content of alkaline ions from foods that are supplied to the body as a result of metabolic processes. The hypothesis of the “acid-ash” diet is to reach a greater alkaline load through a higher consumption of fruit and vegetables and a moderate consumption of proteins [104,105]. It is essential to keep in mind that proteins, after being metabolized, release acid (H^+^). The amount of acids released is related to the type of amino acids they contain. In fact, amino acids can be divided into neutral (alanine, phenylalanine, glycine, isoleucine, leucine, methionine, proline, tryptophan, valine, asparagine, glutamine, serine, threonine, cysteine, and tyrosine), acidic (aspartate and glutamate), and alkaline (arginine, histidine, and lysine). Specifically, lysine, arginine, and histidine form hydrochloric acid, while cysteine and methionine are converted to sulfuric acid. On the contrary, fruit and vegetables, after being metabolized, produce alkali species that are able to neutralize acids [20]. Moreover, foods containing phosphorus, both of natural origin or from food additives, further increase the acid load introduced with the diet [106]. In particular, the suggestions that can be provided to the CKD patients are the following: (i) the consumption of vegetables such as kale, broccoli, brussel sprouts, cabbage, onions, garlic, celery, zucchini, lettuce, cucumber, radish, bell pepper, rocket, and sprouted seeds are all encouraged because of their negative PRAL. It is recommended to include: (i) two portions (about 250 g per day) of the above-cited vegetables in two meals per day; (ii) two portions of fruit (about 300 g per day) after being reviewed according to serum potassium content; (iii) legumes such as lentils, beans, and chickpeas as an alternative source of protein instead of meat; and (iv) meal/grain foods such as bread, breakfast cereals, rice, pasta because of their low PRAL. However, consumption should be limited due to their association with weight gain. It is interesting to reflect on the role of fresh citrus fruit juices in the process of dietary alkalinization. Their effect on the acid–base status is driven by the presence of citrate, a derivative of citric acid. When dietary citrate is absorbed in the intestine thanks to the consumption of citrus juices, it undergoes metabolization into bicarbonate which alkalinizes the urinary pH [73].

An AD for CKD patients can be constructed according to the PRAL and potassium content of different foods. Several studies highlighted that an increase in the consumption of fruit and vegetables in combination with the neutralization of the dietary acid load with the alkali is able to reduce the progression of CKD, monitored by kidney injury biomarkers [59,107]. It is hypothesized that an increase in acid load induces the CKD progression by angiotensin II and endothelin increase [108,109]. Consequently, the activation of the RAAS contributes to the acceleration of CKD progression, causing hyperfiltration and hypertrophy of the residual nephrons with tubule-interstitial damage and glomerulosclerosis. This process is further amplified by the chronic low-grade inflammatory state and oxidative stress, conditions common in CKD patients [110]. A further advantage of the AD in CKD patients is the positive modulation of gut microbiota, inducing an increased production of short-chain fatty acids (SCFAs) and a lower release of nephrotoxic substances, such as p-cresol and indoxyl sulphate [111].

In conclusion, the AD represents an alternative nutritional treatment for CKD patients.

### 3.3. Low-Protein Diet

The LPD is defined by K/DOQI 2020 guidelines [15] as a nutritional approach characterized by a protein intake comprised between 0.55 and 0.60 g/kg of b.w per day, in 3–5 CKD stage without diabetes, metabolically stable under conservative therapy. This nutritional approach should be under close clinical supervision [112].

According to the 2020 K/DOQI guidelines [15], each gram of proteinuria should be replaced in order to maintain the neutral-nitrogenous balance. Usually, in stages IIIb-V of CKD patients, the nutritional approach is based on an LPD characterized by replacing the main carbohydrates sources (such as bread and pasta) with protein-free products that allow the maintenance of a daily protein intake of around 0.6 g/kg of b.w. [14,16,76,113]. It is recommended to “metabolically stable” patients with or without diabetes who are not in RRT. A “metabolically stable” patient has a good glyco-metabolic control, does not present active infections, inflammatory diseases, or cancer, and does not present significant non-intentional short-term weight loss [15]. The principal risk of an unbalanced LPD is the onset of PEW syndrome. The term PEW was firstly defined by the International Society of Renal Nutrition and Metabolism to characterize a series of catabolic and nutritional alterations, which should be present in CKD patients. The causes of PEW syndrome are low caloric intake related to a reduction in appetite and dietary restrictions, a hypercatabolic state typical of CKD, a chronic low-grade inflammatory state, and metabolic acidosis. Altogether, these factors increase the muscle catabolism aggravating uremic sarcopenia [25,114]. In particular, in vivo studies demonstrated that CKD stimulates muscle proteolysis whilst inhibiting lysosomal and calcium-activated proteases. The authors demonstrated that muscle proteolysis would appear to be preventively blocked by administering sodium bicarbonate to rats, highlighting that proteolysis is activated by metabolic acidosis. Proteolysis seems to involve the ATP-dependent ubiquitin-proteasome pathway [46]. In fact, in CKD patients, the correction of metabolic acidosis would seem to decrease protein degradation, amino acid oxidation and inflammatory state, and increase albumin synthesis, impacting positively on their nutritional status [115].

In patients with CKD not complicated by metabolic acidosis, LPD induces a series of adaptive metabolic processes that lead to reduced protein turnover with a consequent decrease in protein-muscle degradation in order to maintain a nitrogen balance. Therefore, during a LPD, it is important to correct metabolic acidosis through oral sodium bicarbonate supplementation or reducing dietetic PRAL, as metabolic acidosis could decrease the effectiveness of the dietary regimen and at the same time accelerate the loss of lean body mass [116].

In order to avoid the onset of PEW syndrome, when setting an LPD, it is necessary that the caloric intake is adequate. For this reason, it is recommended that subjects < 60 years have a caloric intake of 35 kcal/kg of b.w. per day, while subjects ≥ 60 years of 30 kcal/kg of b.w. per day. These nutritional indications can be modified according to patients’ anthropometric characteristics and according to their basal metabolism [117,118].

Regarding LPD efficacy, several metanalysis reported that the lower the protein intake is, the lower the risk of progression to ESRD [119,120,121,122]. Moreover, some studies showed that protein overload is directly correlated to the reduction in the GFR [119,120] and metabolic acidosis [121]. In fact, the latter, as previously highlighted, implements a series of adaptive responses by the kidney aimed at increasing acid excretion involving hormones such as angiotensin II and endothelin, related to more sudden CKD progression [66]. Moreover, both a low protein intake and an improvement in serum bicarbonate levels are associated with a significant reduction in all-causes mortality in CKD patients [123].

Another type of protein restriction diet is represented by the very low-protein diet (VLPD). This nutritional approach is characterized by a protein intake of 0.3 g/kg of b.w. per day, supplemented with essential amino acids and ketoanalogues. The dietary proteins are made up of 90% of vegetable origin. This dietary regime is also characterized by low phosphorus intake (300–450 mg per day) and low salt content (80–85 mmol per day) [124]. The caloric intake must not be lower than 30–34 kcal/kg of b.w. per day. This nutritional regimen seems to improve PRAL and NEAP with beneficial effects on metabolic acidosis [124,125]. An interesting study conducted by Garenata et al. investigated the efficacy of a VLPD supplemented with ketoanalogues compared to a LPD in a stage IV CKD patients. At the end of the study, the VLPD supplemented with ketoanalogues group demonstrated lower renal function loss, compared to the LPD group. Moreover, they showed a better acid–base balance control requiring less treatment with sodium bicarbonate compared to the LPD group. These results suggest that VLPD supplementation with ketoanalogues is a valid nutritional tool to counteract GFR decline and to ameliorate the acid–base balance control [126].

Another study revealed that protein intake in CKD patients appears to be inversely related to serum total CO_2_, a parameter that tends to be reduced in a directly proportional manner to GFR reduction. The impact of dietetic protein intake on this parameter is only evident in CKD patients, while in subjects with normal renal function, the nutritional impact is minimal [127]. The VLPD delays CKD progression by correcting metabolic acidosis and reducing the production of ammonium by the residual nephrons, as the ketoanalogues exert a nephroprotective action. Ketoanalogues protect the kidney against tubulo-interstitial damage induced by increased ammonium production which is normally observed during CKD [128].

As previously mentioned, an alteration in the gut microbiota composition is observed during CKD. In this regard, the LPD would seem to exert a beneficial modulation. Lai et al. conducted a study which compared the traditional LPD with LPD plus inulin, a prebiotic organic also present in protein-free foods. Both diets do not induce qualitative changes of the gut microbiota, but only quantitative changes of the pre-existent species. In particular, the authors observed an increase in Bifidobacteriacae in LPD plus inulin diet group. Bifidobacteria are symbiotic bacteria with a predominantly saccharolytic metabolism which enhance SCFAs production, ameliorating glucose, and lipid metabolism, reducing serum uric acid levels and inflammation and oxidative stress biomarkers, such as C-reactive protein (CRP), tumor necrosis factor (TNF)-α, and NADPH oxidase (NOX)-2. This study confirms that both diets lead to the improvement of metabolic acidosis [129].

LPD and VLPD represent valid nutritional approaches in the management of more advanced stages of CKD. When prescribing these dietetic treatments, it is important to evaluate the correct energy intake because an unbalanced LPD or VLDP can induce malnutrition and PEW syndrome.

### 3.4. Vegan Low-Protein Diet

According to literature data, the LPD represents a cornerstone in the treatment of CKD. In fact, as previously discussed, the main objectives of this nutritional treatment are to avoid toxins accumulation and metabolic acidosis. Amongst LPDs, it is possible to set up different nutritional protocols such as the “conventional” LPD and the vegan LPD. It is not always possible to establish a traditional LDP, for instance if the patient cannot accept using protein-free products, it is possible to resort to a vegan LPD. The vegan LDP is a nutritional approach based on the protein intake comprised between 0.6–0.7 g/kg of b.w. per day. This model is able to provide all essential amino acids in order to guarantee the necessary daily protein intake. In fact, the vegan LPD exploits the complementarity between amino acids of cereals and legumes. For example, if cereals are low in lysine content and high in methionine content, legumes contain high levels of lysine and low levels of methionine; for this reason, a balanced mixture of these foods provides the correct intake of amino acids, according to the recommended daily allowance (RDA) [130]. Evidence has shown that a diet with plant-based proteins favors the hemodynamics in the glomerulus [131]. In fact, Kontessis et al. [131] demonstrated that, regardless of the amount of proteins, plant-based proteins exerted a protective action on renal function. In particular, plant-based proteins seem to prevent glomerular vasodilatation and proteinuria induced by meat consumption. This protective effect should be ascribed to the different hormonal responses induced by plant-based proteins which lower glucagon release and lower renal prostaglandins production, compared to diets with a high meat consumption [132,133]. A further advantage ascribable to plant-based proteins is due to their content of glutamic acid, proline, phenylalanine, cysteine, and serine, which also have a positive impact on renal function [134,135]. Moreover, plant proteins induce a reduction in triglycerides, low-density lipoprotein (LDL) cholesterol, total cholesterol, and uric acid [136]. It is also important to remark that lower amounts of oxidized LDL reduce glomerular damage and CKD progression [91]. In addition, plant-based proteins contain high levels of minerals such as calcium, magnesium, and vitamin C that lower the acid load ameliorating kidney function [137]. Mirmiran et al., highlighted that diets with a high PRAL were associated with a higher prevalence (up to 42%) of CKD onset [137]. The effects induced by the acid load of the diet on renal function are attributable to the increase in ammonia levels in the renal medulla up to determining impaired intratubular and tissue ammonia concentrations, which can negatively impact on tubulo-interstitial damage, determining the progression of CKD [138,139]. Moreover, metabolic acidosis has been associated with an enhanced endothelin production which, in turn, is able to affect GFR decrease [140]. A further mechanism due to a high acid load is ascribable to the high production of oxygen-free radicals and to the increased oxidative stress induced by metabolic acidosis [141].

In this context, a vegan LPD should be a valid therapeutic treatment to decline the progression of CKD.

## 4. Conclusions

Metabolic acidosis represents a serious complication of CKD. As well as being a risk for CKD progression through the increased activation of the RAAS, it tends to aggravate the general clinical conditions of CKD patients by negatively impacting bone and muscle metabolism, whilst exacerbating the progression of renal dysfunction. Therefore, treating metabolic acidosis during CKD becomes fundamental for the optimal clinical management of these patients. Several clinical studies have highlighted how different dietary-nutritional approaches (such as MD, AD, LPD and vegan LPD) represent a valid therapeutic tool able to counteract metabolic acidosis related to CKD.

Preliminary data have demonstrated how different nutritional regimens seem to be equivalent to traditional medical therapies in terms of their ability to correct metabolic acidosis. Moreover, these nutritional approaches are virtually free from side effects.

At present, further randomized clinical trials conducted on larger populations would be necessary to confirm the efficacy of the above-mentioned dietary plans.

## Abbreviations

AD	Alkaline diet
b.w.	Body weigh
BCM	Body cell mass
CKD	Chronic kidney disease
CRP	C-reactive protein
CVD	Cardiovascular disease
DXA	Dual x-ray absorptiometry
ECM	Extracellular mass
ESRD	End stage renal disease
EVOO	Extra virgin olive oil
FGF23	Fibroblast growth factor 23
GFR	Glomerular filtration rate
IGF-1	Insulin-like growth factor 1
IMD	Italian Mediterranean diet
IMOD	Italian Mediterranean organic diet
K/DOQI	Kidney-disease outcome quality initiative
KDIGO	Kidney disease: improving global outcomes
LDL	Low-density lipoprotein
LPD	Low-protein diet
MD	Mediterranean diet
MPCs	Minor polar compounds
MUFA	Monounsaturated fatty acid
NCDs	Non-communicable diseases
NEAP	Net endogenous acid production
NOX	NADPH oxidase
PEW	Protein-energy wasting
PRAL	Potential renal acid load
PTH	Parathyroid hormone
PUFA	Polyunsaturated fatty acid
RAAS	Renin–angiotensin-aldosterone system
RDA	Recommended daily allowance
ROS	Reactive oxygen species
RRT	Renal replacement therapy
SCFAs	Short-chain fatty acids
SFA	Saturated fatty acid
SGLT2	Sodium/glucose cotransporter 2
TNF	Tumor necrosis factor
VLPD	Very low-protein diet
